# A step-wise community engagement and capacity building model prior to implementation of mhGAP-IG in a low- and middle-income country: a case study of Makueni County, Kenya

**DOI:** 10.1186/s13033-018-0234-y

**Published:** 2018-10-15

**Authors:** Victoria N. Mutiso, Isaiah Gitonga, Abednego Musau, Christine W. Musyimi, Eric Nandoya, Tahilia J. Rebello, Kathleen M. Pike, David M. Ndetei

**Affiliations:** 1 Africa Mental Health Research and Training Foundation, Matumbato Road, Off Elgon Road, Mawensi Gardens, Nairobi, Kenya; 2 Global Mental Health Program, Columbia University, New York, USA; 3 University of Nairobi, Nairobi, Kenya

**Keywords:** mhGAP-IG, Stakeholders, Mental health, Implementation, Community, Kenya

## Abstract

**Background:**

The World Health Organization developed the Mental Health Gap Action Programme Intervention Guide (mhGAP-IG) as guidelines for innovative utilization of available resources in low- and middle-income countries (LMICs) in order to accelerate the reduction of the mental health treatment gap. The mhGAP-IG calls for each country to contextualize the guide to their social, cultural and economic context. The objective of this paper is to describe a model for a stepwise approach for implementation of mhGAP-IG in a rural Kenyan setting using existing formal and informal community resources and health systems.

**Methods:**

We conducted an analysis of mental health services in Makueni County, one of the 47 counties in Kenya, in order to understand the existing gaps and opportunities in a low-resource setting. We conducted stakeholder analysis and engagement through interactive dialogue in order for them to appreciate the importance of mental health to their communities. Through the process of participatory Theory of Change, the stakeholders gave their input on the process between the initiation and the end of the process for community mental health development, with the aim of achieving buy-in and collective ownership of the whole process. We adapted the mhGAP-IG to the local context and trained local human resources in skills necessary for the implementation of mhGAP-IG and for monitoring and evaluating the process using instruments with good psychometric properties that have been used in LMICs.

**Results:**

We were able to demonstrate the feasibility of implementing the mhGAP-IG using existing and trained community human resources using a multi-stakeholder approach. We further demonstrated the feasibility to transit seamlessly from research to policy and practice uptake using our approach.

**Conclusions:**

An inclusive model for low resource settings is feasible and has the potential to bridge the gap between research, policy and practice. A major limitation of our study is that we did not engage a health economist from the beginning in order to determine the cost-effectiveness of our proposed model, occasioned by lack of resources to hire a suitable one.

## Background

A major barrier to addressing the high burden of mental disorders in LMICs [1] is the lack of adequate resources and the non-equitable distribution of resources (mainly human and financial) dedicated to mental health services [2] and to reducing stigma [3]. To address these issues, WHO developed the mhGAP-IG (v.1) [4] through a process of rigorous international expert consensus [4]. The mhGAP-IG is a tool designed to assist resource-limited settings in their efforts to scale up the coverage of mental health services for their citizens and in the process reduce the treatment gap [5]. The mhGAP-IG is designed for use by non-mental health specialists, focuses on selected priority mental and neurological disorders (MNDs), and provides a systematic framework for recognition and provision of evidence-based interventions, both pharmacological and non-pharmacological for treating these conditions. The priority conditions are depression, psychosis, dementia, bipolar disorder, epilepsy, behavioral disorders, developmental disorders, alcohol use disorders, drug use disorders, suicide and self-harm. WHO recommends contextualization of the generic version of the mhGAP-IG be implemented in each country/local context in order to produce a fully adapted version that meets the needs of the existing health system in which it is to be used [4]. A recent systematic review has established the wide-spread application of the mhGAP-IG across multiple LMICs [6].

### The Kenyan context

In Kenya, only 0.05% of the National Health budget goes to mental health [7], mainly to support operational costs for psychiatric hospitals or units. The overall national health budget does not differentiate mental illness from non-communicable or communicable diseases. However, Kenya has taken the initiative to tackle mental health, evidenced by the launch of the mental health policy on May 17th 2016 [8] which recognized the need to provide mental health services to all Kenyans up to the community level and integrated with other health services. It is hoped that this will lead to more allocation of funds, given the high disability burden caused by mental disorders in particular depression [9]. For instance, our previous studies found the prevalence of depression in Kenya varies from 18.7% in household surveys [10] to 42% in populations attending general health facilities [11]. Up to 96% of cases of depression in populations in general health facilities go unrecognized [11].

In addition to the poor funding for mental health [7], there are few mental health specialists, lack of awareness on mental disorders and stigma [3, 10, 12], all contributing to the existing large mental health treatment gap, to the extent that most of those in need of mental health services are unable to receive quality care [13]. In Kenya, health services are devolved to the 47 counties, who then prioritize the services according to their own context and needs while the Ministry of Health at national level gives overall guidance on county polices and technical advice.

There are two main health service systems in Kenya, as described below:

(i) *The Informal health sector* This sector is composed of Traditional Healers (TH), Faith Healers (FH) and community health volunteers (CHVs). Many people with mental illness consult THs and FHs as their first line of treatment, especially in more rural communities [14], though people use a combination of THs/FHs and the formal health sector [15].
  There is increasing evidence that THs and FHs can play vital roles in the delivery of mental health care with proper training, supervision, support and there is a precedent of them making referrals to the formal sector [16]. There is also evidence that it is possible for the THs, FHs and the formal sector to come together to dialogue on shared values and common ground [17]. THs and FHs are important component/primary resource to be harnessed in local healthcare plan. In India, THs and FHs are the first contact in the pathway to care for the majority of psychiatric patients [18]. In a meta-analysis and systematic review [19], it was found that about half of the individuals seeking formal health care for mental disorders in Africa choose traditional healers (48.1% 95% CI 36.4–60.0) and religious/faith healers (49.2% 95% CI 38.0–60.4) as their first provider.
  Community Health Volunteers (CHVs) are also part of the informal health sector and are the backbone of community-based programs on HIV/AIDS, TB, nutrition, and health education [18, 19]. CHVs have successfully provided most essential lifesaving interventions, especially those among the leading causes of child mortality [20–22]. They also equip families with the knowledge and skills to prevent disease. They promote good nutrition, sanitation, hygiene and link families to essential services [22, 23].
  Previous studies by Africa Mental Health Research and Training Foundation (AMHRTF) [24, 25], other studies in Kenya [26] and from and other LMICs [27] have suggested that they can be trained to provide mental health awareness and screen for mental disorders. CHVs link health centers and dispensaries with the surrounding communities. They are recruited by the communities in conjunction with the Departments of Health (DoH) at the County level. They are usually high school graduates who receive training by the DoH on home visits and community follow-up through a community health approach system.
(ii) *The Formal sector* The formal health sector mostly offers conventional health services based on the “western” allopathic model. This sector includes health facilities at six levels: Level 1—community; Level 2—dispensary; Level 3—health centre; Level 4—Sub-County hospital; Level 5—County referral hospital and Level 6—national referral hospital. Level 1 is at the community level which is the focus of service delivery priorities and includes villages, households, families and individuals. Levels 2 and 3 provide mostly promotion and prevention-based care, while levels 4 and above address curative and rehabilitative services and to some degree promotive and preventive activities [28]. Doctors and specialists are only found in levels 4, 5 and 6. Levels 1, 2 and 3 have nurses and clinical officers and include health centers, dispensaries and community-based purveyors of care.

Though Kenya has approximately 100 psychiatrists for a population of 45 million (ratio 1:450,000), which is the best in the region [29], it still falls below the recommended minimum psychiatrist to population ratio of 1:10,000 [30]. These psychiatrists are found mainly in level 6, episodically in level 5 and mainly work in private practice (for which they charge for their services) in large urban areas. They are relatively inaccessible to the majority who need mental health services due to geographical distance to the urban areas and unaffordable consultation fees.

### The Makueni County context

**Fig. 1:**
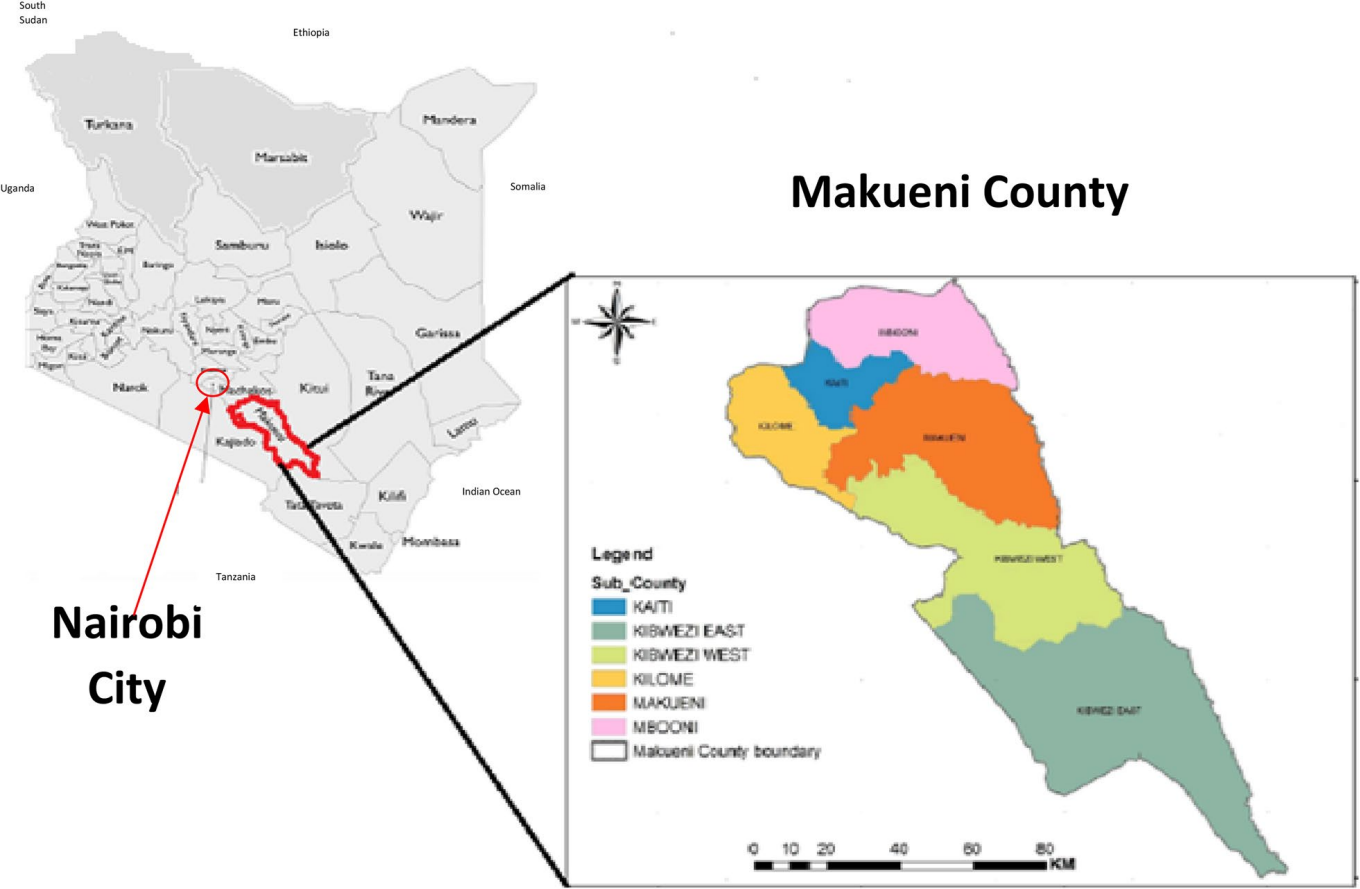
It demonstrates the location of Makueni County in relation to the capital city of Nairobi and the rest of the counties. It also demonstrates the size and the administrative regions of Makueni County

The geographical context of Makueni County in relation to other Counties is summarized in Fig. 1. It is located 250 km southeast of Nairobi extending across the busy Mombasa-Nairobi Highway, and is comprised of 6 Sub-Counties. It is sparsely populated with about 1 million residents (51% female and 49% male), a population density of 124.9 people per km^2^ and a total of 186,478 households (an average of 5.4 members per household). It has one of the poorest economies in Kenya [31] with over half (65%) of the population living on less than 1US dollar per day [32]. The economy is mainly subsistence agriculture, i.e. crop farming and keeping livestock to support family needs.

The County has the following healthcare facilities: A County referral hospital, 6 Sub-County hospitals, 21 health centers, 113 dispensaries and 11 private clinics. The health centres and dispensaries were allowed to stock psychotropics on the Essential Medicines List 2010 [33], which included diazepam, phenobarbital and chlorpromazine (injectable). However the essential drugs list represents only the minimum that was mandatory. We were informed during the meetings with county health officials that the county was not prohibited to stock other medicines according to any newly identified needs and demands.

The bed capacity in the county stands at 616 i.e. one bed for 1623 people compared with the national average of 1.4 beds per 1000 people [34] and the doctor population ratio is 1:22,712 [35] which is below the accepted standard of 9:100,000 [29]. There is neither a psychiatrist nor a clinical psychologist. However, there are five psychiatric nurses, all of whom were approaching retirement. Due to the overall shortage of health personnel, these psychiatric nurses also perform other general nursing and administrative duties, making them less available for provision of psychiatric services [36].

There are nine Voluntary Counselling and Testing (VCT) clinics with 138 counsellors. The average household distance to a health facility is 6 km which is below the national recommended distance of 4 km [35]. In spite of these, it was the first county in Kenya to advocate for mental health at the policy level and to fund mental health services using public funds as a result of a partnership with AMHRTF to match funds to support this study.

### Preliminary findings of a pilot project on task shifting in mental health in Makueni County

From the preliminary but unpublished findings on task shifting (i.e. training non-specialists on skills to provide service normally provided by specialists in a particular service) conducted by AMHRTF at a health centre and one Sub-County hospital in Makueni County in 2008–2009, we learned that task shifting in mental health was feasible and therefore had potential for scale-up. As part of that pilot, 6 health workers (nurses and clinical officers), 6 THs, 6 FHs and several CHVs were trained at the two health facilities mentioned above. We increased the number of referrals to the facilities from zero to over 1500 in about 12 months. The greatest drawback of the pilot was that we did not adopt multi-stakeholder engagement, community ownership and leveraging of available resources and systems for seamless transition to scale and enhancement of sustainability.

In this article, we describe the implementation process of our protocol titled “Multi-sectoral Stakeholder *TEAM* Approach to Scale-Up Community Mental Health in Kenya—Building on Locally Generated Evidence and Lessons Learned (TEAM)” using the experiences of conducting the pilot study on task shifting. We sought to mobilize all existing community resources, identify and engage with relevant stakeholders right from the beginning and to participate in the implementation of the protocol. We hypothesized that this would lead to collective ownership of the whole process and fast tracking the implementation of the emerging policy and practice.

## Methods

This model was implemented in seven stages as discussed below:

### Baseline analysis of mental health systems

In order to understand the existing health system in Makueni County and how mental health was accommodated, we conducted a baseline analysis of mental health systems including formal and informal service providers in the study sites. For this, we used the World Health Organization Assessment Instrument for Mental Health Systems (WHO-AIMS), for collecting essential information on the mental health system of a country or region [37]. We held discussions with the representatives of the various stakeholders on their perceptions of mental illnesses including but not limited to causes, how they presented, what should be done and stigma. We also scrutinized medical and inventory records. This exercise allowed us to have an up-close view of the existence and deficiencies in the following elements on mental health: policies, outpatient and inpatient services, integration into primary healthcare, available human resource, public education and inter-sectoral links research such as data collection as well as monitoring and evaluation activities. (The details of how we conducted the WHO-AIMS, the findings and recommendations are under consideration for publication in a different manuscript).

### Engaging stakeholders

We engaged representatives of the following stakeholders: Policy makers including direct engagement with the Governor and Deputy Governor of Makueni County, the senior Department of Health officials at the County Government level, formal and informal service providers, pharmaceutical technicians and records officers at each of the healthcare facilities, community opinion leaders or gatekeepers (described below under bottom up approach), representatives of the Association of Users and Survivors of Psychiatry in Kenya (USP-Kenya) at national and local levels and a social and anthropological scientist. However, we could not engage a health economist due to limited resources. Stakeholders were involved at several phases: (1) Development of the concept for the TEAM grant application—we consulted and obtained support from the Governor, other department of health officials; (2) Planning for implementation; (3) Continued engagement throughout the implementation and validation of the findings for joint and communal ownership, including engagement of policy makers especially during the intervention period. The points of discussions were informed by our findings on WHO-AIMS (published separately).

We took two approaches to stakeholder engagement: A bottom-up and top–bottom approach to obtain community and political leaders’ buy-in and ownership of the process and participation in Theory of Change (ToC) as described below.

(i) *The bottom-up approach* We aimed to effectively leverage existing human, financial and infrastructural resources without overburdening them or creating a parallel system and without undue demand on community and government resources i.e., we did not demand for extra resources except leveraging on existing resources. Key stakeholders were the “community gatekeepers” who are ordinary but trusted community members acting as scouts for the community and privy to private community discussions; some of these gatekeepers are community leaders who are the custodians of the communal cultural norms and values. The importance of working through local leaders, as opposed to only government, administrators, and politicians was informed by our experience from the pilot task shifting study when we realized communities demanded a say in what happened in their localities.
(ii) *Top–bottom approach* This was meant primarily for the policy makers who have the final say on official policies, planning and financing. We did this in several complementary ways: the first was by creating political priority for mental health services by cultivating a community demand for these services so that it takes a position on the political agenda that elected politicians cannot overlook. We did this through stakeholder engagement process and participatory ToC (described below) workshops. Secondly, we engaged the Governor, County politicians, Minister for Health and County Health Management Teams. These are the custodians of health policies and practice at the County level. Through engagement with the Governor and his senior officials as part of the top–bottom approach, we specifically aimed to strengthen relationships between the research team and the County’s top leadership and secure their cooperation in advance. We also sought their facilitation of actual project implementation in the following areas: a pledge by the Governor to commit government funds to the project through a matching fund (to support procurement of required psychotropic medicines, in-kind support for the nurses and clinical officers to provide services, training support and facilitation of county employees involved in mental health) and a lobby for waiver system for all fees to patients for mental health services offered at the public facilities where this project was to be implemented. We brought up the concept of mental health being fully integrated into the health system in the facilities at which the project was to be implemented.

AMHRTF is already a recognized member of the County stakeholders providing specialized consultation in mental health. This gave AMHRTF a good opportunity for engagement and therefore paired a top–bottom approach with the bottom-up approach.

The following were also consulted: (1) An expert on the human rights of people with mental illness from the National Council of Persons with Disability (NCPD); (2) local and national leadership of THs and FHs; (3) Users and Survivors of Psychiatry (USP), the national user movement. We maintained continuous engagement and dialogue with the stakeholders through regular progress reports and with the service providers through face-to-face support and supervision every two weeks and telephone support as needed.

### Participatory development of Theory of Change (ToC)

ToC is a specific type of methodology for planning, participation and evaluation that has been widely used in different sectors to promote social change [38]. The ToC is very appropriate in that it makes the distinction between desired and actual outcomes and requires all stakeholders to model their desired outcomes before they decide on forms of intervention to achieve those outcomes [39]. ToC is derived from the critical theory [40]. ToC has been used successfully in various disciplines [41], in government and non-government entities across both developed and developing countries [42]. In 2002, it was fronted as the best model for comprehensive community initiatives evaluation [43].We chose participatory ToC because it allows all participating stakeholders to agree upfront what they want to achieve and then work backwards collectively to map the pathway to impact. ToC has been used to develop and evaluate health initiatives in various LMICs in mental health [38, 40, 44] including Kenya [45].

In the course of stakeholder engagement, we shared with them the WHO-AIMS findings and the gaps between the ideal and current states. Prior to the implementation, we held dialogues with them on what was ideal (i.e. what we wanted to achieve and the assumptions) and asked for their inputs, as summarized under results. Together with the stakeholders, we discussed how these gaps could be bridged by identifying barriers, suggested solutions, facilitators/enablers and how to identify them.

### Customizing mhGAP-IG: Adaptation and adoption of mhGAP-IG in the context of Makueni County

The aim of the adaptation of the mhGAP-IG was to make it context-appropriate for Makueni County for two purposes: (1) to enhance community awareness by the various stakeholders so that they clearly understood the symptoms of the various disorders in order to facilitate and promote health seeking behavior; (2) to serve as a more applicable identification and clinical intervention tool by the trained nurses and clinical officers.

The adaptation process: The mhGAP-IG was meant for situations just like Kenya and had been piloted by WHO before they recommended it for LMICs [4] but also recommended local adaptation and gave guidelines within the document, which we followed. We customized all the sections of the mhGAP-IG (v.1) and included all the seven sections for each of the specified priority conditions including assessment, management (psychosocial and biological i.e. use of psychotropic drugs but only by nurses and clinical officers), follow up and referrals. The adaptation process was done by a team that included a psychiatrist; three clinical psychologists; three psychiatric nurses, three clinical officers and a public health physician who met for 5 full days, each day covering no more than two priority conditions. They had back and forth discussion on the mhGAP-IG intended meaning of each of the symptoms of each of the priority conditions until a consensus or common understanding was achieved on the concepts. The adapted English version was given to a linguist who translated into the local dialect and another person translated it back to English version. There was back and forth translation until the final English version agreed with the adapted version. We rearranged the adapted version as follows: (1) The symptoms of each of the priority conditions were extracted for use to train THs, FHs and CHVs, so that they could then use them to create awareness in the communities. They could also use them to screen (using the mhGAP-IG master chart) and refer those who screened positive to the nearest health facility with trained research assistants, who could then administer confirmatory tests and make referrals to trained nurses and clinical officers as needed. (2) The whole adapted document was used to train the nurses and clinical officers. They were to make referrals only to the nearest County hospital for cases they did not feel competent enough to manage. These were then piloted with 5 each of the following drawn from outside the study area but within Makueni County: FHs, THs, CHVs, nurses and clinical officers. They were then adopted for the study. A later stage customization involved inclusion of psychotropic drugs not in the list of essential drugs but were later deemed necessary depending on clinical situations during the actual implementation of the adapted version of mhGAP-IG.

### Review and culturally-sensitive translation of data collection tools for the monitoring and evaluation of TEAM

We used a researcher designed socio-demographic questionnaire and the tools described below which have documented good psychometric properties, have been validated for use by lay interviews and have been used extensively in LMICs including other studies in Kenya. We translated these using the same team and same method we used for the mhGAP-IG and then piloted them before use.

(i) *WHO’s Quality of Life*-*BREF tool* (QoL-BREF) [46] for impact on self-reported quality of life, functioning, wellbeing, and economic outcomes; (ii) *Self*-*Stigma of Mental Illness* [47] to measure stigma; (iii) *mhGAP*-*IG suicidality symptoms* to assess suicidal ideation/behavior; (iv) treatment compliance survey to assess treatment adherence; (v) *WHO intimate partner violence* (IPV) *scale* to assess changing patterns of reported gender-based violence. We included intimate partner violence (IPV) primarily to determine its prevalence in the local context and to inform future interventions [48]; (vi) *WHO Disability Assessment Schedule 2.0* (WHODAS 2.0) [49], (vii) Alcohol, Smoking, Substance Involvement Screening Test (ASSIST) for screening alcohol and substance use disorders [50]; (viii) Patient Health Questionnaire (PHQ-9) for screening, diagnosing, monitoring and measuring the severity of depression [51], (ix) Researcher-designed socio economic status and health questionnaire; (x) *The M.I.N.I. International Neuropsychiatric Interview* (M.I.N.I. 6.0) for psychiatric evaluation and outcome tracking [52]; (xi) *Mini*-*Mental State Examination* (MMSE) used to measure cognitive impairment [53]; (xii) *Washington Early Recognition Center Affectivity and Psychosis* (WERCAP) screen used to identify clinical risk for developing bipolar or psychotic disorders [54] and (xiii) Seizure calendar for monitoring seizures in epileptic patients [55]. By definition, none of the above instruments is necessarily a purely clinician-administered instrument but all can be administered by trained lay interviewers by simply reading the questions to the interviewee without any interpretation up to three times or can be self-administered.

The purpose of specifying the tools we used is to demonstrate the feasibility of their administration by trained research assistants (RAs), drawn from the community and therefore the possibility for their use for inbuilt longitudinal monitoring and evaluation of the scaled-up community mental health service program. Different countries and contexts have the choice to determine what instrument to use to suit their needs. The instruments were adapted for Makueni County by convening a meeting of key stakeholders and individuals with relevant experience. The meeting comprised of a psychiatrist, clinical psychologists, a public health physician, nurses, medical anthropologists, sociologists and residents from Makueni County who were conversant and fluent with the native Kamba language. We reviewed each tool to ensure terminologies were all culturally appropriate, context specific and retained intended meaning. The adaptation, translation, piloting and adoption process was similar to that described for the mhGAP-IG. All these tools had been used in the past by AMHRTF in research in other parts of Kenya. It was therefore much easier to adapt them for Makueni, with each adaptation session for each tool lasting up to 60 min.

### Facilitate community mental health awareness

It has been demonstrated that awareness and psychoeducation increases health seeking behavior, compliance and results in better treatment outcomes [56–58]. The various trained health providers were asked to conduct awareness campaigns through health talks with their clients. CHVs conducted mental health interactive talks (i.e. audiences asked questions) as follows: (i) at least one public meeting in the communities across the 20 target healthcare facilities (ii) at least one session to patients at the waiting bay in the 20 healthcare facilities. (iii) During any of their contacts with patients during their routine contacts for other conditions. The talks focused on symptoms and the various diagnosis in the WHO mhGAP-IG; that mental disorders are illnesses for which there are effective treatments like any other illness, with the difference that they are surrounded by superstition, stigma and discrimination which were the major barriers to treatment; that people with mental illness had the right to access treatment as part of their human rights; and that we had trained the nurses and clinical officers in their catchment area and were ready to provide the services. Our specific request was that if they recognized any of the symptoms in their clients they should advise referral to the nearest health facility or advise the clients and their friends to refer themselves if they had such symptoms.

### Training of human resources

#### Training of health providers, pharmacists and health records officers

Focusing on skills and competencies rather than on theory is what the mhGAP-IG recommends for non-mental health specialists [59]. All the trainings were done by a team of mental health experts from AMHRTF comprised of a Ph.D clinical psychologist specializing in community mental health, a PhD nurse specializing in global mental health, a public health physician and supervised by the psychiatrist on the research team. We used the adapted and adopted mhGAP-IG covering all the priority disorders. We trained on a maximum of two conditions per one full day focusing on skills rather than theory. The total priority conditions therefore took five full working days. The FHs, THs and CHVs were trained on how to use the various symptoms to create awareness and to screen for the various symptoms for adult disorders as listed in the mhGAP-IG then use standard prescribed referral form developed by the research team to refer to the nearest health facility where there were trained RAs, nurses and clinical officers. They were not trained on interventions. Nurses and clinical officers were trained on how to diagnose the various disorders according to the symptoms listed in the mhGAP-IG and also how to provide both psychosocial interventions and biological (psychotropics) interventions. They were trained on following psychotropics which the County Government provided for free and were to be supervised and supported by AMHRTF: Carbamazepine, sodium valproate, amitriptyline, fluphenazine, fluoxetine, chlorpromazine (tablets), haloperidol (both tablets and injectable), lithium (only to be endorsed by a psychiatrist from AMHRTF team) and diazepam. This list is richer than the one in the mhGAP-IG with the County Government pledging to supply any other psychotropics, if needed. After the initial training, research team from AMHRTF made field in person supervisory visits every 2 weeks and telephone supervision anytime according to need. They were also trained to make referrals, if necessary, to the hospital as detailed in the mhGAP-IG. Training consisted of PowerPoint presentations, case vignettes, small group works and several role-plays on each of the diagnostic area modules. The role plays were very relevant in promoting interactions within the group, building confidence in their competence to recognize different symptoms and disorders, make referrals, and for the nurses and clinical officers to provide appropriate interventions. The pharmacists and pharmaceutical technicians were trained on psychotropic drug administration and how to maintain stocks according to demand, current and anticipated. The record officers were trained on how to record any mental health disorder(s) diagnosed using the MINI-Plus alongside any co-morbid diagnosis being managed on the same patients, thus creating an integrated health information system.

#### Training of research assistants (RAs) before the start of the project—Planning for in-built monitoring and evaluation

We recruited and trained 20 high school graduates identified from the community as RAs who worked within the healthcare facilities that we had selected for the implementation of mhGAP-IG. RA trainings focused on the efficient and accurate administration of the psychometric instruments listed above, including the socio-demographic questionnaire. We used group training to increase inter- and intra-rater reliability. This included group discussions, role-plays, and mock administrations of the instruments on each other and then observations on one of the experts from AMHRTF administering the instruments to real volunteer patients. They were trained on obtaining informed consent. They were also trained on how to administer psychometric instruments and MINI-Plus for DSM-IV/ICD 10 diagnosis. For all psychometric instruments they were to record the questions the way they were without interpreting them and record the answers. If the participant did not understand, they were allowed to repeat the same question up to two times making a possible maximum of three times for all participants. There was special emphasis on emergency referrals for suicidal behavior, psychosis and disruptive behavior according to the mhGAP-IG. The trained RAs were posted to each of the 20 study sites. All the psychometric instruments were administered on all the referred patients on arrival at the health facility and conducted in a quiet room provided by the facility for this purpose. This was preceded by informed consent obtained by the trained RA. With the exception of socio-demographic and IPV questionnaires which were administered only at baseline and before any intervention, all other instruments were administered at baseline, just before intervention and at every subsequent follow up. Only patients who had been referred following the community awareness and training of THs, FHs, CHVs, nurses and clinical officers or were self-referrals or referrals by families/friends were included in the study.

All participants who received a confirmatory DSM-IV/ICD-10 diagnosis by the independent RA were referred to mhGAP-IG trained nurses and clinical officers, working in an environment in which they had the necessary psychotropic drugs, fully paid for by Makueni County. These trained health professionals received further support and supervision by mental health specialists from AMHRTF, through bimonthly face-to-face meetings, and through telephone supervision, as required. The option for higher referrals and laboratory support was also made available when needed.

## Results

The focus of this paper is not on patient outcomes of interventions but the approach to developing integration of community mental health services. Detailed patient outcomes are being subjected to different publications. Thus, the results presented below focus on process and implementation outcomes.

1. *Baseline analysis of mental health system* the main finding was that there was a dearth of mental health services across all health sector levels. The existing health systems catered almost exclusively for non-mental health conditions. These gaps were further highlighted during ToC described below.
2. *Customizing mhGAP*-*IG adaption and adoption of mhGAP*-*IG to the context* There were no contentious issues during the customization of mhGAP-IG. We now have a customized mhGAP-IG that can be used by primary healthcare workers to create awareness on mental health symptoms and disorders; can be used as a screening tool for the priority conditions in the mhGAP-IG by primary healthcare workers and to provide psychosocial and biological interventions by nurses and clinical officers.
3. *Stakeholder engagement* the main results of this exercise were: (1) the mapping of community resources; we identified and engaged with different formal and informal health providers that were subsequently trained to enhance mental health awareness. (2) A multi-stakeholder participatory ToC in place (Fig. 2). (3) Through regular briefs we stayed engaged with policy makers, community opinion leaders and other leaders and non-health sector disciplines. The net effect was connected communities focused on mental health.

**Fig. 2:**
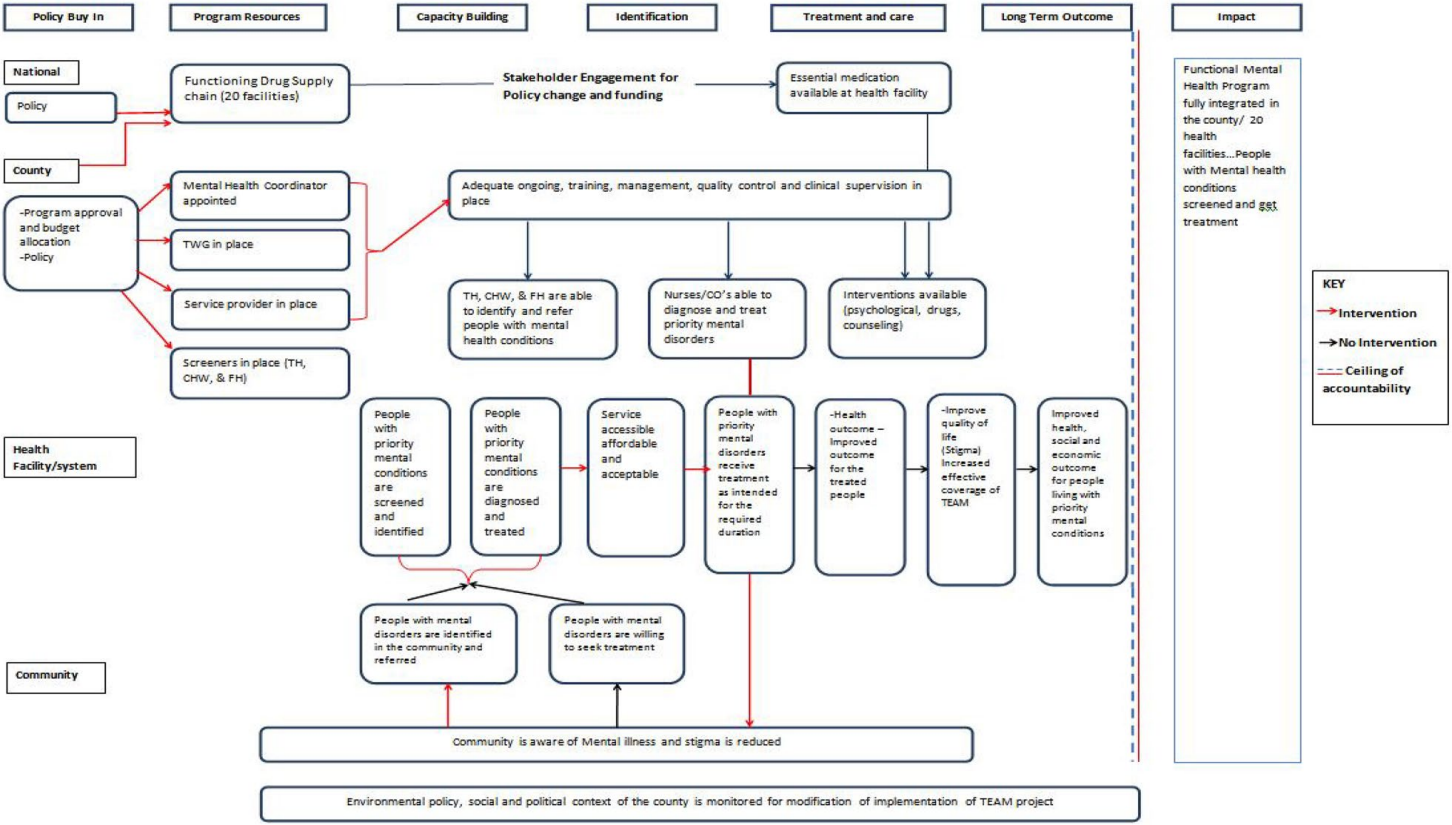
It is the Theory of Change (ToC) developed through interactive multi-stakeholder participation. It is a summary of the activities and building blocks that link existing health system and resources and how to harness them towards a functional and integrated community mental health program that results in communities accessing mental health services
4. P*olicy maker engagement and evolving policy* Through engaging policy makers as key stakeholders, we strengthened relationship between the research team and the County Governor and his senior health officials. Even before the actual implementation of the project, the Governor pledged to commit government funds to the project i.e. matching funds. This was to come in supporting procurement of required psychotropic medicines, support and facilitation of training of county employees involved in mental health, and a pledge by the county to fully sponsor the training of a psychiatrist and a psychiatric clinical officer as well as support in the provision of mental health services. The Governor also provided a waiver system for all fees to patients for mental health services offered at public facilities. Mental health was fully integrated into the health system in the facilities at which this research was done.
  An important policy change was expansion of availability of psychotropics by the Makueni County Government beyond the essential list to meet the newly identified needs and demands. These needs and demands arose during the implementation of mhGAP-IG in order to treat the identified DSM-IV/ICD 10 diagnoses. This justified and allowed for stocking of the following additional psychotropics (some of which previously had only been allowed at hospital level): Carbamazepine, sodium valproate, amitriptyline, fluphenazine, fluoxetine, citalopram, haloperidol (both tablets and injectable) and lithium carbonate. These were fully paid for by the Makueni County Government as their contribution to the study.
5. *A participatory Theory of Change* The components of ToC are summarized in Fig. 2. The highlights of key recommendations on pathway to success are summarized.
  **(**a) Indicators of success: (i) increased number of people receiving appropriate treatment; (ii) improvement in health, social and economic outcomes of people living with mental disorders; (iii) increased availability of mental health services (b) Functions of health facilities: (i) ensure medication supply chain is functional (make orders); (ii) detect/screen and assess for priority mental health disorders; (iii) improve case detection in the community; (iv) provide mental health intervention (drugs and/or counselling). (c) Assumptions: (i) Committed leadership at national, county and health facility levels; (ii) Trained health workers remain in the healthcare facilities or county and on-job training continues for new and more health staff; (iii) THs/FHs are taking up referral roles without fear or intimidation.
6. *Health workers trained* We trained the following on the mhGAP-IG: 20 nurses; 20 clinical officers; 59 THs, 51 FHs and 60 CHWs. We also trained 20 pharmacist’s assistants on psychotropics; and 20 record officers on merging health report systems to include mental health. All those trained were equitably distributed across 20 health facilities.
7. *Research assistants trained* One for each facility trained on consenting and administration of psychometric instruments and for confirming DSM-IV/ICD10 diagnosis using the MINI Plus.
8. *Monitoring and evaluation* We demonstrated the feasibility of using several psychometric instruments by trained RAs from the community to monitor and evaluate the study outcomes. In the process we demonstrated significant positive changes (p < 0.05) in various social, psychological and clinical outcomes and critical numbers seeking and accessing mental health service (all of these are being published separately).

## Discussion

This is the first Kenyan model for a community, stakeholder and multi-disciplinary collectively-owned approach to implementation of mhGAP-IG, using already available resources with the potential for sustainability and scale-up. Of significance is the joint participation of stakeholders and community members in mapping out a potential pathway, including collectively identifying and negotiating predetermined goals, barriers and enablers. The Government of Makueni leveraged mental health services into existing services to accommodate mental health without compromising on other services. This allowed for seamless and timeless transition from research to policy and practice, where the stakeholders collectively own the science, policy and practice. As a pilot, TEAM was located in one of the 47 counties in Kenya and has the potential to be tried out in the remaining counties. Although we cannot assume complete uniformity across all 47 counties, we assume more similarities than differences. We also assume the same for other similar socio-cultural and economical settings.

This model demonstrates that it is feasible to identify and bring together various national and community human resources (formal and informal) including; government officials, human rights activists, patients, their families and other community stakeholders to work in synergy as partners and build community social capital focusing on mental health using the WHO mhGAP-IG model, ratified by Government of Kenya as a member state of WHO. As expected, there were no contentious issues in the customization of mhGAP-IG as it was designed, tested and recommended for use in such contexts. It is significant that the informal sector and, in particular, THs and FHs made referrals to the healthcare facilities. Their ability to participate and contribute to ToC means they had the capacity to understand what needed to be achieved, and how to handle challenges that emerged. The success in training RAs, drawn from the community and their ability to administer all the tools suggests the feasibility of continuous monitoring and evaluation of the community mental health services. Moreover, by consulting other disciplines, there was diversity on views of mental health service delivery, but more importantly, a consensus on approach in the context of Makueni County, the first time it happened in Makueni County.

The final product was a collective ownership of the process from inception. An important result was the continuous policy evolvement and development as part of the process and part of the integration of mental health services into the existing services at the facilities where AMHRTF provided training. These evolving policy changes are summarized under “*Policy maker engagement and evolving policy*”, section #4 of the results.

On a long-term basis, Makueni County has already started to fully sponsor training of specialists in mental health (i.e. psychiatrists, counseling psychologists, psychiatric nurses, and psychiatric clinical officers).

This study is unique compared with similar mhGAP-IG programs in LMICs. For instance, the PRogramme for Improving Mental health carE (PRIME) study only did comparative analysis of mental health care plans components and human resource requirements in five countries [58] while studies in Nigeria [60] and Ethiopia [61] did not involve the informal service providers. None of these studies took a comprehensive approach for collective ownership of the entire process. However, there is a cross-cutting theme that calls for contextualization which may vary from country to country and similar settings. TEAM provides a comprehensive and inclusive model that can be used in other similar situations including similar LMICs. Where there are no pilot studies, unlike ours where there was an earlier pilot study, the WHO-AIMS is a readily available generic tool for understanding the context or for enriching any pilot studies like in our case. The extent of monitoring and evaluation and therefore the choice of psychometric tools will vary according to the needs of particular settings.

This study adds value to the collective body of data and experiences in the implementation of the mhGAP-IG [6]. According to our research, this is the first reported mhGAP-IG implementation study that has the widest inclusivity, spectrum of activities, measures that were undertaken and reported in a single study. It is also the first study to demonstrate implementation research in mhGAP-IG that has inbuilt and simultaneous policy and practice, creating a seamless integration of research, policy and practice.

As the wave of Global Mental Health takes root in both high-income countries and LMICs and looks forward to partnership and collaboration between South and South and between North and South, it is critical for all parties to understand and appreciate the intricacies of different contexts, and that no model is universally suitable especially if the focus is to be on the most underserved, resource-poor rural communities with no mental health specialists.

We attribute the feasibility and apparent success of our model to collective multi-stakeholder team approach, mutually respectful dialogue where every opinion and health system (formal or informal) count and, in particular, policy makers’ engagement right from the beginning and staying engaged with them to the end. Our model demonstrates the feasibility to implement mhGAP-IG in LMICs using already existing resources and therefore sustainable.

A major limitation is that this project was piloted in only one of the 47 counties in Kenya. However, the primary health care system is fairly uniform across all the 47 counties which, until the new constitution in 2010, were all under one national government. As such, the model described here is amenable to adoption by the other counties in Kenya. The only non-routine, available human resource was the training of RAs. However, it is to be noted that the RAs were recruited from the same community and therefore part of community capacity building. For lack of resources, we did not include a health economist from the beginning to end to advise in advance necessary prospective data collection for determination of cost-effectiveness.

## Conclusions

Subject to the limitations highlighted under discussion, our model demonstrates the following:

(1) Despite the existing resource challenges, LMICs can mitigate the mental health treatment gap today, while working to improve the resources for tomorrow. (2) Collective ownership, multi-stakeholder and multi-disciplinary approaches in the process of developing and implementing community mental health services are critical. (3) Traditional policy briefs at the end of the studies, with the possibility of having them ignored or shelved, should be relegated to a process of participatory involvement of policymakers and the continuous policy development and evolution. (4) Implementation of mhGAP-IG needs to be contextualized using currently available resources, and it is feasible. The details will vary from one context to another.
